# Low-Dose Atropine 0.025% for Childhood Myopia Control: A Retrospective Review in Clinical Practice

**DOI:** 10.3390/jcm15218587

**Published:** 2026-11-04

**Authors:** Ng Kaiwen, Aaron Qi Yang Goh, Bryan Xiangrong Sim, Sri Yudah, Pauline Poh Lin Chan, Hla Myint Htoon, Audrey Wei Lin Chia

**Affiliations:** 1 Ministry of Health Holdings Private Limited (MOHH), Singapore 139691, Singapore; ng.kaiwen@mohh.com.sg (N.K.);; 2 Singapore National Eye Centre (SNEC), Singapore 168751, Singapore; bryan.sim.x.r@singhealth.com.sg (B.X.S.); sri.yudah@snec.com.sg (S.Y.); pauline.chan.p.l@snec.com.sg (P.P.L.C.); 3 Myopia Centre at Bedok, Singapore 460212, Singapore; 4 Singapore Eye Research Institute (SERI), Singapore 169856, Singapore; hla.myint.htoon@seri.com.sg

**Keywords:** myopia, atropine, combination

## Abstract

**Aim:** In this retrospective audit, Atropine 0.025% eyedrops were evaluated in a clinical setting. **Method:** Children aged 4–16 years, who were started on Atropine 0.025% with at least 6 months of follow-up, were included. A total of 588 eyes (299 children) were classified as treatment-naïve eyes started on Atropine 0.025% daily (Group A, n146), eyes progressing on Atropine 0.01% daily increased to 0.025% daily (Group B, n287) or combined with myopia control glasses (Group C, n66), and those stable on Atropine 0.01% twice daily converted to Atropine 0.025% daily (Group D, n89). Spherical equivalent (SE) and axial length (AL) were recorded from 6 months before treatment to 24 months after treatment began. **Result:** Group A children were younger (8.4 ± 1.5 years), while Group D children were older (10.6 ± 1.59 years). Progression in the first 6 m was significantly less than that in 6 m prior in Groups A-C. At 1 year, a change in AL < 0.15 mm was noted in 41%, 49%, 50% and 35%, and a change in AL ≥ 0.30 mm was observed in 38%, 18%, 32% and 23% of eyes in Groups A, B, C and D, respectively. Treatment was increased in 37%, 34%, 12% and 41% of eyes over 2 years in Groups A, B, C and D, respectively. Mean progression at 2 years was −0.63 ± 0.86 D or 0.44 ± 0.31 mm, −0.39 ± 0.59 D or 0.30 ± 0.26 mm, −0.30 ± 0.48 D or 0.36 ± 0.40 mm, and −0.48 ± 0.54 D or 0.33 ± 0.22 mm in Groups A, B, C and D, respectively. Multivariate analysis suggested that progression was mainly associated with younger age. **Conclusions:** Atropine 0.025% was a useful addition to myopia control, with some eyes requiring further modification in treatment.

## 1. Introduction

Axial or School Myopia, characterised by excessive axial length (AL) elongation, is the leading cause of correctable vision loss worldwide [1]. It is estimated to affect 50% of the global population by 2050 and continues to increase in prevalence, especially in the East Asia region [1,2]. This poses significant public health concerns due to the association of high myopia with visual complications such as myopic macular degeneration (MMD), choroidal vascularisation (CNV), and retinal detachment [3,4,5]. Fortunately, there are now several treatment options available to slow myopia progression, including Atropine eye drops, myopia control glasses and contact lenses [6].

Atropine, a non-selective muscarinic antagonist, has been used for decades as a treatment modality to slow myopia progression [7,8,9]. Initially, high concentrations of Atropine (0.5–1.0%) were shown to be efficacious in slowing the progression of myopia [7,8,9]. However, more recent studies suggested that lower doses of Atropine (0.01–0.1%) were also effective in slowing myopia progression, with fewer side effects such as glare and near blur due to pupil dilation and accommodation loss, respectively [10,11]. Response is dose-related, and clinicians have been adding more doses to their clinical practice to provide greater options for patients.

The aim of this study was to describe the use of Atropine 0.025% either alone or in combination with myopia control glasses in a clinical setting.

## 2. Methods

In this retrospective audit, children (aged 4–16 years) started on Atropine 0.025% (Myopine 0.025%, Xepa-Soul Pattinson (M) Sdn Bhd, Melaka, Malaysia) daily at the Singapore National Eye Centre between June 2021 and May 2023 were reviewed. We excluded patients with ocular diseases (e.g., cataract, amblyopia, or strabismus), allergy to Atropine, previous use of myopia control glasses or contact lenses, poor compliance with Atropine treatment, and those with less than 6 months of follow-up after starting Atropine 0.025% eye drops.

Participants were then divided into 4 groups based on the treatment they received. Group A comprised treatment-naïve eyes which were started on Atropine 0.025% daily. Group B comprised eyes progressing by >0.5 D per year on Atropine 0.01% daily and were escalated to Atropine 0.025% daily. Group C comprised children progressing by >0.5 D per year on Atropine 0.01% daily and increased to a combination of Atropine 0.025% and myopia control glasses, either the Highly Aspherical Lenslet Target (HALT) or Defocus Incorporated Multiple Segments (DIMS) glasses. Group D comprised children who were quite stable on Atropine 0.01% twice daily and were converted to Atropine 0.025% once daily.

The primary outcome was myopia progression, assessed by the rate of changes in spherical equivalent (SE) and axial length (AL) from baseline at 6, 12, and 24-month periods.

Demographic data including age, gender, and ethnicity were collected for each participant. Data for SE and AL were collected at 5 time points: pre-Atropine 0.025% (defined as 6–12 months prior to starting Atropine 0.025%), at baseline, and at 6, 12, and 24 months after starting Atropine 0.025%. For refractive error, cycloplegic refraction was prioritised. Cycloplegic refraction was obtained 30 min after administration of 2 drops of cyclopentolate, spaced 5 min apart. If this was not available, non-cycloplegic refraction was used instead. On occasions where vision was good (i.e., 6/7.5 or better), refraction was not always done, and the lensometer reading of current glasses was used. SE was calculated using the following formula: spherical power plus half of cylinder power. AL was measured by partial coherence interferometry using the IOL Master 500 (Carl Zeiss Meditec AG, Jena, Germany).

Any change in treatment over time was documented. Treatment was deemed to have increased if there was any increase in dose or frequency of eye drops, or addition of optical myopia control glasses or contact lenses (where they were not used previously). Conversely, it was deemed to have been decreased if the dose or frequency of eye drops were decreased or an optical treatment was removed (where it had been used previously). It was deemed to have been swapped if an equivalent dose was used (e.g., if Atropine 0.025% daily was reduced again to Atropine 0.01% twice daily). Presence of any adverse effects (e.g., blurred vision, glare, or itch) was documented.

As children may not be seen at exact time points, measurements within 3 months were collected for each 6-month time point. Change in SE and AL was calculated as change from baseline divided by follow-up time and multiplied by time point. For example, if a change of −1.00 D was collected at 13 months, then change at 12 months was (−1.00/13) × 12 or −0.92 D.

Demographic data (i.e., gender, race and age) and primary outcome measures (baseline SE and AL, and change in SE and AL at 6 months prior to treatment and at 6, 12, and 24 months after treatment) were computed within each treatment group. Progression of SE and AL at all time points, despite changes in treatment, was assessed, equivalent to an intention-to-treat model. Progression in whom treatment was changed and unchanged was also evaluated. Counts were presented as numbers and percentages, while continuous variables were described with means and standard deviation. Differences between groups were assessed using generalised estimating equation (GEE) models to account for the longitudinal and clustered use of both eyes in the same subject over time. Differences within groups were assessed using regression models after clustering eyes by subjects. *p* values of <0.05 were considered significant. Univariate and multivariate analyses were conducted to explore potential associations for greater myopia progression. All analyses were performed using STATA 11 BE (StataCorp LLC, College Station, TX, USA).

## 3. Results

Five hundred and eighty-eight eyes (299 children) were included and categorised into four groups: treatment initiation (Group A; *n* 146), increment (Group B; *n* 287 eyes), combination (Group C; *n* 66 eyes), and conversion (Group D; *n* 89 eyes). Ten children had treatment started in only one eye, and one child had different treatments started in each eye. A refractive error reading was present in 100%, 96% and 83% of eyes, but cycloplegic refraction was only done in 50%, 19% and 4% at baseline and at 1 and 2 years, respectively (Supplemental Figure S1). An axial length measure was recorded in 80%, 75% and 74% at baseline and at 1 and 2 years, respectively.

**Figure 1:**
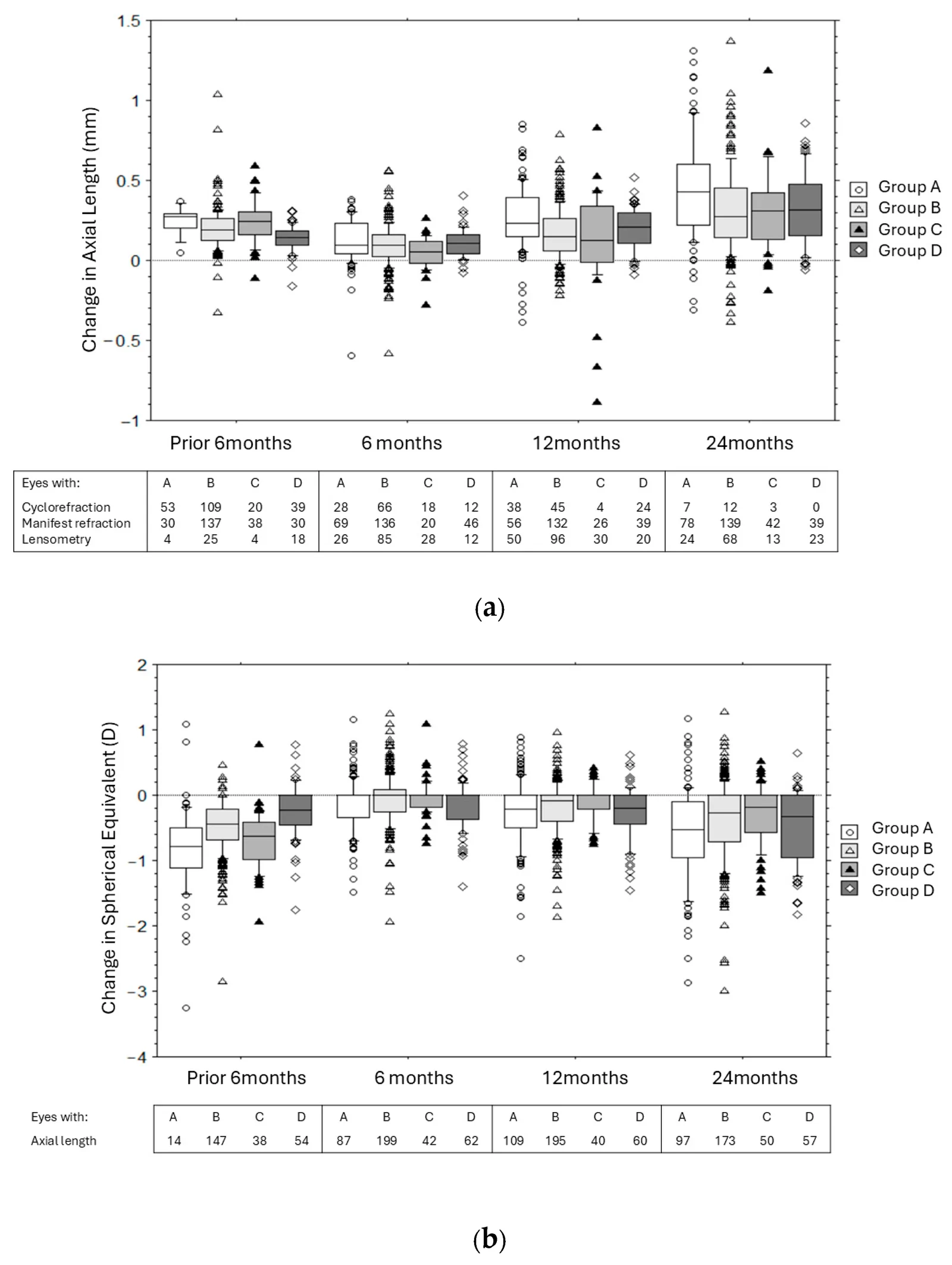
(**a**) Change in spherical equivalent (D) in the 6 months prior to treatment and at 6, 12 and 24 months of treatment in eyes in different groups. (**b**) Change in axial length (mm) in the 6 months prior to treatment and at 6, 12 and 24 months in eyes in different groups. Group A: treatment-naïve eyes started on Atropine 0.025% daily; Group B: children progressing on Atropine 0.01% daily converted to Atropine 0.025% daily; Group C: children progressing on Atropine 0.01% daily converted to Atropine 0.025% daily combined with myopia control glasses; and Group D: children on Atropine 0.01% twice daily converted to Atropine 0.025% daily.

Children in Group A tended to be younger (8.4 ± 1.5 years) with lower myopia, while those in Group D were older (10.6 ± 1.9 years) with higher myopia (Table 1). The estimated change in SE 6 months prior to baseline was greatest in Group A, followed by Groups C and B, and least in Group D (Table 2, Figure 1a). The mean change in AL 6 months prior was quite similar in Groups A-C and less in Group D. Six months after baseline, there were significant reductions in SE and AL progression in Groups A, B and C, while no significant change was noted in Group D.

Treatment was altered in 41%, 36%, 15% and 45% of eyes in Groups A, B, C and D over 2 years (Table 2; Supplemental Figure S1). Overall, myopia progression was greatest in Group A, followed by Groups D, B and C (Table 2; Figure 1a,b).

At 1 year, progression by <0.25 D was noted in 54%, 64%, 69% and 57%, and progression by ≥0.5 D was noted in 25%, 17%, 10% and 21% of eyes in Groups A, B, C and D, respectively. A change in AL < 0.15 mm was noted in 41%, 49%, 50% and 35% of eyes in Groups A, B, C and D, respectively; a change in AL ≥ 0.30 mm was noted in 38%, 18%, 32% and 23% in Groups A, B, C and D, respectively.

By 2 years, progression by <0.5 D was noted in 49%, 61%, 71% and 55%, and progression by ≥1.0 D was noted in 21%, 12%, 9% and 23% of eyes in Groups A, B, C and D, respectively. A change in AL <0.3 mm was noted in 33%, 54%, 44% and 45% of eyes in Groups A, B, C and D, respectively; a change in AL ≥ 0.6 mm was noted in 25%, 11%, 14% and 12% in Groups A, B, C and D, respectively.

Univariate analysis suggested that greater progression of SE over 1 year might be associated with younger age and a more negative SE, while faster progression of AL might be associated with younger age and greater myopic progression in the 6 months prior to baseline (Supplemental Table S1). Multivariate analysis suggested that faster myopia progression was mainly associated with younger age after controlling for gender, race and treatment group (Table 3).

Glare/photosensitivity was noted in three children, blur in two, and four children reported itching and discomfort. No child stopped treatment because of adverse effects.

## 4. Discussion

The present study explored the clinical use of 0.025% Atropine in four different treatment groups. These groups were quite different from each other, reflecting children at different stages of their myopia journey: from treatment initiation (Group A), to managing continued progression (Groups B and C), to relative stability (Group D). Differences in age, individual propensities for progression and natural slowing of myopia over time might influence the overall change in SE and AL in each group, thus limiting direct comparisons between groups.

Children in Group A were treatment-naïve, and Atropine 0.025% daily was started as a first-line treatment when it became available at our institution. Not unexpectedly, these children were significantly younger than those in other groups (mean age of 8.4 years vs. 9.3–10.6 years). They also had lower myopia at baseline and greater myopia progression in the 6 months prior to treatment (−0.97 ± 1.11 D or 0.24 ± 0.08 mm) compared to 6 months after starting treatment (−0.14 ± 0.40 D, or 0.11 ± 0.14 mm, *p* < 0.001) (Table 2). Over the first year, a change in SE < 0.25 D or AL < 0.15 mm was noted in 54% and 41% of eyes, respectively, while a progression in SE ≥ 0.50 D or AL > 0.30 mm was noted in 25% and 38%, respectively. Treatment was increased (i.e., dose increase or optical treatment added) in 19% and in an additional 26% of eyes by the end of the first and second years, respectively. This increment was likely because of excessive myopia progression, given the change in eyes in which treatment was increased (i.e., −0.41 D or 0.31 mm over first year) was greater than that in eyes where treatment was unchanged (i.e., −0.20 D or 0.23 mm) (Table 2). However, it could occasionally be by choice as alternative treatments (especially optical treatments) were becoming increasingly available during this time.

Three studies have explored the use of Atropine 0.025% in treatment-naïve children. These include a retrospective review in Korean children (mean age of 8.4 years) by Moon & Shin (2018) and two randomised controlled trials: the Low-Concentration Atropine for Myopia Progression (LAMP) Study and ORANGE Study [11,12] which were done in Hong Kong children (mean age of 8.5 years) and Japanese children (mean age of 9.4 years), respectively (Table 4). The SE progression with Atropine 0.025% groups in these studies (mean −0.44 to −0.50 D) was larger, but the AL progression (mean of 0.29 to 0.30 mm) in the first year was similar to eyes in Group A from our study (means of −0.27 D and 0.26 mm). Sample size differences (ethnicity, environment, baseline age and myopia degree) may explain differences in response between different studies (Table 4). The inconsistent use of cycloplegic refraction in this audit could also result in a less reliable SE measure, while AL measurement may remain more objective.

Children in Groups B and C (i.e., those who were still progressing on Atropine 0.01%) were similar in age and SE and AL at baseline, although SE change in the 6 months prior to treatment was greater in Group C than B (Table 2). There was no difference in the change in SE/AL between Groups B and C at 6 months, 12 months and 24 months. Proportionately, treatment modification was also more likely in Group B (36%) than in Group C (15%) over 2 years. Progression in eyes in the first 6 months prior to when treatment was changed was larger in Group B eyes, suggesting that treatment was increased because of faster progression. This suggests that the increase in treatment could blunt progression so that subsequent change in both groups was roughly similar.

Several studies now suggest that combination treatment may be more effective than monotherapy alone. Cao et al. (2024) found that combination therapy with 0.01% Atropine and DIMS lenses resulted in less myopic progression (−0.22 D or 0.16 mm) when compared to 0.01% Atropine monotherapy (−0.58 D or 0.40 mm) in Chinese children over a 12-month follow-up period [14]. Nucci et al. (2023) demonstrated similar effects in European children, where myopia progression over 1 year was −0.40 D or 0.10 mm for children on Atropine 0.01% alone, and −0.02 D or 0.05 mm for children on combination therapy with DIMS-Atropine 0.01% [15].

Finally, children in Group D were previously on Atropine 0.01% twice daily and converted to Atropine 0.025% daily. These children were significantly older with higher myopia at baseline. Myopia progression in the 6 months prior to initiating Atropine 0.025% was much lower than in the other groups, and subsequent reductions in SE and AL were also less marked. This suggests that conversion between doses was possible with no significant loss in efficacy. Indeed, the once-daily dose is more convenient, which could result in better compliance.

Multivariate analysis suggested that myopia progression over 12 months was primarily associated with younger age (Table 3). Clinically, this suggests that younger myopic children could benefit from earlier and more aggressive treatment, and clinicians should be prepared to escalate treatment if required. Other factors such as poor compliance, lack of outdoor activity, increased near work, parental myopia, and higher education levels have also previously been cited as risk factors for poor treatment response to Atropine [11,16,17].

There are several limitations in this study. Due to the retrospective nature of this study, the choice of treatment is subject to selection bias depending on clinical advice, as well as patient/parental choices. When the Atropine 0.025% dose was introduced, clinicians were advised to follow up with children every 6 months, with refraction and axial length measurements performed at each visit. However, there was some variability in the timing of follow-up visits, and cycloplegic refraction and axial length measures were not always available at every visit. In particular, the rate of cycloplegic refraction decreased over time, and accurate unbiased measures of SE cannot be assumed. Calculation of the rate of AL and SE assumes a linear progression over time, which may result in both over- and under-estimation of true progression. Reduction in myopia progression over time may also reflect natural slowing of myopia progression rather than a true drug effect, especially in older children. Children started on different optical treatments were grouped together, and possible differences in the efficacy of individual optical treatments were not accounted for. Compliance at each visit was not consistently assessed.

## 5. Conclusions

Findings from this clinical audit, particularly the changes noted between the 6-month periods prior to and after treatment and progression, suggest that Atropine 0.025% was useful as an introductory dose for treatment-naïve children or where treatment escalation and change were required. However, given the non-random nature of how children were assigned to treatment and changes in treatment over time, the exact extent of the treatment effect cannot be determined with certainty. Future studies may consider extending the follow-up period to assess the long-term safety and efficacy of Atropine 0.025%. Additionally, comparisons between Atropine monotherapy and combination therapy present a meaningful avenue for future research.

## Figures and Tables

**Table 1:** Demographics and baseline spherical equivalent and axial length in different groups.

	Group A (Start)	Group B (inc A0.025)	Group C (inc Combo)	Group D (Swap)	*p*-Value <0.05
Eyes/Subjects	146/74	287/147	66/33	89/46	
Male (*n*, %)	62 (42%)	136 (47%)	30 (45%)	48 (53%)	
Chinese (*n*, %)	125(85%)	235 (81%)	62 (93%)	81 (91%)	
Age (Yrs, mean, SD)	8.4 (1.5)	9.9 (1.7)	9.3 (1.3)	10.6 (1.9)	AB, AC, AD, CD
Spherical Equivalent (D)	−4.07 (2.05)	−4.79 (2.31)	−4.79 (1.81)	−5.83 (2.77)	AD
Axial Length (mm)	24.71 (1.17)	25.14 (1.05)	24.84 (0.88)	25.76 (1.17)	AD, BD, CD

Note: Counts were depicted as number (percentage), while continuous values were presented as mean (standard deviation).

**Table 2:** Change in spherical equivalent and axial length over time.

	Group A (Start)	Group B (inc A0.025)	Group C (inc Combo)	Group D (Swap)	*p*-Value <0.05
Spherical Equivalent (D)					
Change Pre 6 m	−0.97 (1.11)	−0.46 (0.46)	−0.69 (0.45)	−0.22 (0.38)	AB, AD, BC, BD, CD
Change First 6 m	−0.14 (0.40)	−0.07 (0.39)	−0.02 (0.29)	−0.14 (0.35)	nil
-pre vs. first 6 m	*p* 0.001	*p* 0.001	*p* 0.001	*p* 0.244	
Change over 12 m *	−0.27 (0.51)	−0.17 (0.38)	−0.10 (0.26)	−0.26 (0.40)	AC
Change over 24 m *	−0.63 (0.86)	−0.39 (0.59)	−0.30 (0.48)	−0.48 (0.54)	AB
Axial Length (mm)					
Change Pre 6 m	0.24 (0.08)	0.20 (0.14)	0.23 (0.13)	0.13 (0.08)	AD, BD, CD
Change First 6 m	0.11 (0.14)	0.09 (0.13)	0.04 (0.09)	0.11 (0.09)	AC
-pre vs. first 6 m	*p* 0.017	*p* 0.001	*p* 0.001	*p* 0.072	
Change over 12 m *	0.26 (0.20)	0.17 (0.16)	0.18 (0.20)	0.19 (0.12)	AB
Change over 24 m *	0.44 (0.31)	0.30 (0.26)	0.36 (0.40)	0.33 (0.22)	AB
Eyes in which treatment was changed between 6 and 12 m					
Number	28 (19%)	47 (16%)	2 (6%)	26 (29%)	
Increase:Swap:Decrease	26:2:0	47:0:0	2:0:0	26:0:0	
Change SE first 6 m	−0.51 (0.39)	−0.32 (0.47)	−0.05 (0.36)	−0.34 (0.30)	AC, BC
Change AL first 6 m	0.22 (0.12)	0.18 (0.13)	0.10 (0.11)	0.16 (0.07)	AC, BC, CD
Eyes in which treatment was changed between 12 and 24 m					
Number	33 (22%)	57 (20%)	6 (9%)	15 (16%)	
Increase:Swap:Decrease	29:0:4	51:4:2	6:0:0	11:0:4	
Change SE over 12 m	−0.41 (0.47)	−0.25 (0.31)	−0.19 (0.27)	−0.26 (0.32)	nil
Change AL over 12 m	0.31 (0.25)	0.27 (0.12)	0.30 (0.11)	0.21 (0.13)	nil
Eyes which continued on same treatment (i.e., excludes eyes where treatment was changed)					
Change SE over 12 m	−0.20 (0.49)	−0.13 (0.32)	−0.10 (0.26)	−0.17 (0.35)	nil
Change SE over 24 m	−0.28 (0.61)	−0.21 (0.45)	−0.30 (0.48)	−0.28 (0.45)	nil
Change AL over 12 m	0.23 (0.20)	0.15 (0.16)	0.19 (0.20)	0.16 (0.11)	nil
Change AL over 24 m	0.35 (0.26)	0.22 (0.22)	0.38 (0.43)	0.24 (0.19)	nil

* Includes eyes in which treatment was changed between 6 and 24 m.

**Table 3:** Multivariate analysis of change in spherical equivalent (SE) and axial length (AL) over 12 months with different variables (italics indicate significance *p* < 0.05).

	Change in SE over 12 Months			Change in AL over 12 Months		
	Coeff	95% CI	*p*-Value	Coeff	95% CI	*p*-Value
Female (ref male)	−0.00	−0.08, 0.07	0.879	0.01	−0.03, 0.06	0.502
Chinese (ref non-Chinese)	0.12	−0.00, 0.26	0.057	−0.02	−0.04, 0.09	0.547
Age at baseline	0.05	0.02, 0.07	<0.001	−0.04	−0.06 −0.02	<0.001
Baseline SE	−0.00	−0.02, 0.01	0.588	-	-	-
Change SE pre 6 m	−0.01	−0.10, 0.08	0.896	-	-	-
Baseline AL	-	-	-	0.04	0.02, 0.06	<0.001
Change AL pre 6 m	-	-	-	0.25	−0.29, 0.53	0.079
Groups (A -ref)						
B	−0.00	−0.13, 0.13	0.992	−0.06	−0.16, 0.03	0.219
C	0.08	−0.05, 0.23	0.236	−0.06	−0.19, 0.05	0.285
D	−0.14	−0.31, 0.02	0.094	−0.01	−0.11, 0.09	0.783

**Table 4:** Comparison of studies exploring the use of low-dose Atropine in clinical practice.

	Study Population	Dose	Change in SE and AL
This study, SG	Retrospective. N299, mean 9.6 Y (4–16 YO), SE −4.6 D	A: start A0.025	1 Y −0.27 D/0.26 mm; 2 Y −0.63 D/0.44 mm
		B: Inc to A0.025	1 Y −0.17 D/0.17 mm; 2 Y −0.39 D/0.30 mm
		C: Inc to combined	1 Y −0.10 D/0.18 mm; 2 Y −0.30 D/0.36 mm
		D: swap to A0.025	1 Y −0.26 D/0.19 mm; 2 Y −0.48 D/0.33 mm
Comparison with other studies with Atropine 0.025%			
Moon 2018, Korea [12]	Retrospective. N285, mean age 8.2 YO (5–14 YO), SE −3.9 D	Pre-treatment	−1.6 D/y or 0.55 mm/y
		A0.01	−0.8 D/y or 0.44 mm/y
		A0.025	−0.5 D/y or 0.30 mm/y
		A0.05	−0.2 D/y or 0.22 mm/y (converted from m)
Yam 2019/20 = LAMP, HK [11]	RCT. N 438, mean age 8.5 YO (4–12 YO), SE −3.8 D	Placebo	1 Y: −0.81 D/0.41 mm
		A0.01	1 Y: −0.59 D/0.36 mm; 2 Y: −1.12 D/0.59 mm
		A0.025	1 Y: −0.46 D/0.29 mm; 2 Y: −0.73 D/0.50 mm
		A0.05	1 Y: −0.27 D/0.20 mm; 2 Y: −0.55 D/0.39 mm
Ohno-Matsui 2026 = ORANGE, JP [13]	N 299, mean age 9.4 YO (5–15 YO), SE −3.08 D	Placebo	1 Y −0.95 D; 2 Y −1.65 D/0.74 mm
		A0.01	1 Y −0.66 D; 2 Y −1.31 D/0.64 mm
		A0.025	1 Y −0.44 D; 2 Y −1.02 D/0.51 mm

LAMP: low-concentration Atropine for myopia progression; HK: Hong Kong; JP: Japan; SG: Singapore; RCT: randomised control trial; SE: spherical equivalent; AL: axial length; Y: year; m: month; YO: year old.

## Data Availability

The original contributions presented in this study are included in the article. Further inquiries can be directed to the corresponding author.

## Supplementary Materials

The following supporting information can be downloaded at: https://www.mdpi.com/article/10.3390/jcm15218587/s1.
