# Supplementary material for: Low-Dose Atropine 0.025% for Childhood Myopia Control: A Retrospective Review in Clinical Practice
Source: J Clin Med. 2026 Nov 4;15(21):8587. doi: 10.3390/jcm15218587 (PMC13764573; doi:10.3390/jcm15218587)
Supplement: Supplementary file 1 [file jcm-15-08587-s001.zip › jcm-4578935-supplementary.pdf]

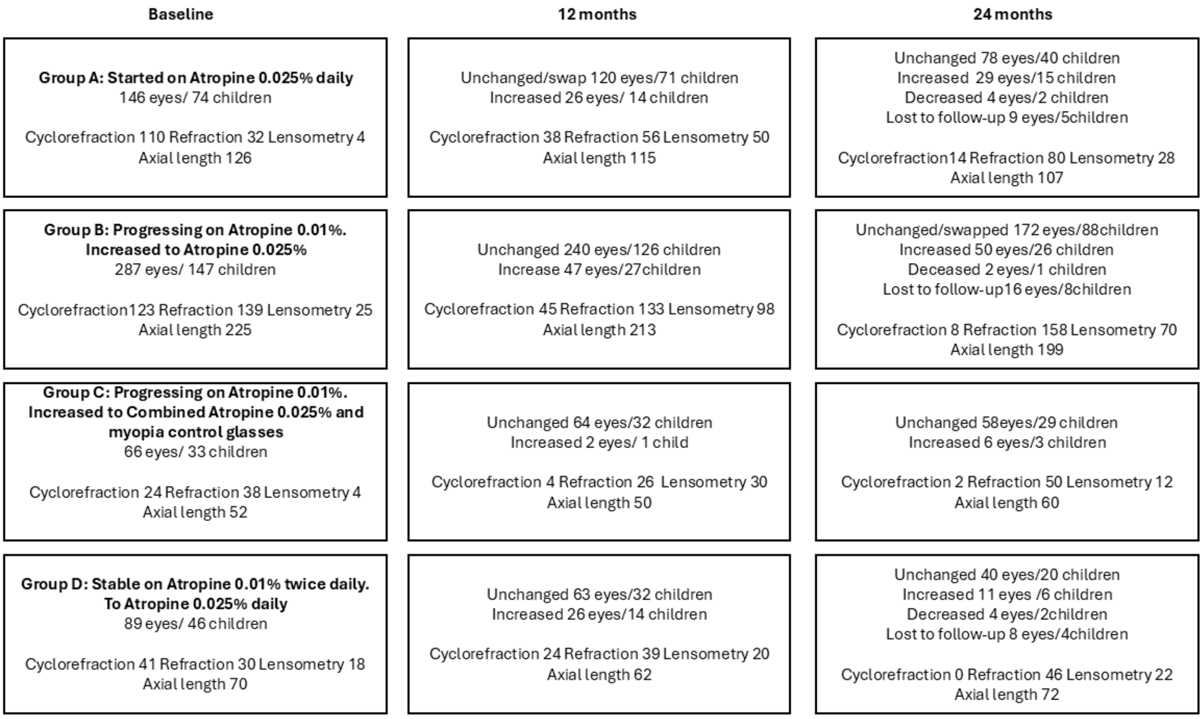

Supplemental Figure S1.

Note: Sum of children may be more than expected in each category as some children had treatment in only 1 eye, different treatments started in both eyes, or treatment changed at different time in each eye. Note numbers may differ from Figure 1 and 2 as it will require measures from 2 time points to assess change.

Supplemental Table S1: Univariate analysis of demographic and ocular parameters with change in spherical equivalent (SE) and axial length (AL) over 12m.

	**Change of SE over 12m**			**Change of AL over 12m**		
	Coeff	95% CI	p-value	Coeff	95% CI	p-value
Female (ref Male)	0.01	-0.06, 0.10	0.714	-0.01	-0.05, 0.03	0.652
Chinese (ref Non-Chinese)	0.09	-0.03, 0.23	0.146	-0.01	-0.08, 0.05	0.650
Baseline age	0.05	0.02, 0.07	*<0.001*	-0.03	-0.04, - 0.02	*<0.001*
Baseline SE (D)	-0.02	-0.04, -0.01	*0.013*	0.00	0.00, 0.01	*0.039*
Change SE pre 6m	0.01	-0.10, 0.11	0.900	-0.07	-0.11, -0.20	*0.004*
Baseline AL (mm)	0.01	-0.02, 0.05	0.494	0.00	-0.01, 0.02	0.662
Change AL pre 6m	-0.42	-0.85, 0.01	0.053	0.36	0.05, 0.66	*0.020*
Group A (ref)						
Group B	0.09	-0.01, 0.21	0.101	-0.09	-0.14, -0.33	0.002
Group C	0.17	0.04, 0.29	0.007	-0.07	-0.16, 0.01	0.096
Group D	0.00	-0.13, 0.15	0.901	-0.06	-0.12, 0.01	0.052
